# Tongue Rhabdomyosarcoma in Adults: A Case-Based Review

**DOI:** 10.7759/cureus.86192

**Published:** 2025-06-17

**Authors:** Giorgos Sideris, Panagiotis P Gogoulos, Theofanis Nastos, Vasileia Damaskou, Petros V Vlastarakos

**Affiliations:** 1 Second ENT Department, Attikon University Hospital, National and Kapodistrian University of Athens, Athens, GRC; 2 Second Pathology Department, Attikon University Hospital, National and Kapodistrian University of Athens, Athens, GRC

**Keywords:** adult, pleomorphic rhabdomyosarcoma, radiation therapy, surgical resection, tongue

## Abstract

Rhabdomyosarcoma (RMS) of the tongue in adults is an exceptionally rare and aggressive malignancy, often posing significant challenges in diagnosis and management. Due to its rarity, most available data come from isolated case reports and small retrospective studies. It typically presents as a rapidly enlarging tongue mass, leading to symptoms such as dysphagia, speech disturbances, and airway obstruction. Diagnosis relies on imaging studies and histopathological confirmation, with immunohistochemical markers playing a crucial role in differentiation from other malignancies. We present a case of an adult patient who developed a tongue mass with progressive symptoms. Imaging revealed a large tumor at the base of the tongue, and biopsy confirmed a high-grade RMS. Due to worsening clinical symptoms, surgical resection was performed before the final histopathological diagnosis was available. Despite initial treatment, the tumor recurred, requiring a second surgical intervention followed by radiation therapy. At the 12-month follow-up, the patient remained disease-free. A literature review identified 12 previously reported cases of adult tongue RMS, highlighting the variability in treatment approaches, with most patients undergoing surgical resection, often combined with chemotherapy or radiotherapy. Given the high risk of recurrence, a multimodal treatment strategy is necessary to improve survival outcomes. Early diagnosis and regular follow-up are critical as recurrence often necessitates additional surgical interventions. Further studies are needed to better understand the long-term prognosis and optimize treatment strategies for this rare malignancy.

## Introduction

Rhabdomyosarcoma (RMS) is a rare and aggressive malignant tumor that originates from mesenchymal cells committed to skeletal muscle differentiation [1]. Although it primarily affects children, accounting for approximately 5% of all pediatric cancers and representing the most common soft tissue sarcoma in this population, it can also occur in adults, albeit far less frequently [2]. Among adults, RMS is associated with a poorer prognosis due to delayed diagnosis, more aggressive subtypes, and limited treatment data.

RMS may arise in any anatomical region but has a predilection for the head and neck, genitourinary tract, and extremities [3]. Within the head and neck region, involvement of the oral cavity, and particularly the tongue, is exceedingly rare in adults [3-4]. The tumor typically presents as a rapidly enlarging mass, and in the case of lingual RMS, it may lead to symptoms such as dysphagia, odynophagia, speech impairment, or ulceration. Regional lymph node involvement and distant metastases are not uncommon, reflecting the tumor’s aggressive biological behavior.

Histologically, RMS is classified into four main subtypes: embryonal (more common in younger children, generally favorable prognosis), alveolar (more aggressive, often in adolescents), pleomorphic (mainly in adults, aggressive), and spindle cell/sclerosing (rarer, with variable outcomes) [1,5-6]. Understanding these subtypes is essential for diagnosis, prognosis, and therapeutic planning.

The diagnosis of RMS is challenging due to its rarity in adults and non-specific clinical presentation. It requires a high index of suspicion, supported by imaging studies and confirmed via histopathological and immunohistochemical analysis. Misdiagnosis or delayed diagnosis can lead to advanced disease at presentation, affecting treatment outcomes.

In this report, we present a rare and clinically significant case of RMS located at the base of the tongue in an adult patient. This case is noteworthy due to the extreme rarity of lingual RMS in adults, its challenging diagnostic process given the tumor's nonspecific presentation and overlapping features with more common benign or inflammatory lesions, and the potential for serious functional impairments, including effects on speech, swallowing, and airway patency. By detailing the clinical presentation, diagnostic workup, imaging findings, surgical considerations, and therapeutic course, we aim to contribute valuable insights to the limited body of literature on adult tongue RMS and support earlier recognition and improved management of similar cases in the future.

## Case presentation

A 49-year-old male with a history of neurofibromatosis type 2 presented to the emergency department with a three-month history of halitosis. He was also complaining of progressively worsening dysphagia along with a foreign body sensation, a “hot potato voice,” and over the last month. He denied dyspnea, respiratory distress, weight loss, or disturbed sleep patterns. Upon further inquiry, the patient stated that he had been able to tolerate both solids and liquids until recently and had adapted by consuming softer foods. He maintained normal sleep without requiring positional adjustments or supportive measures such as sleeping in an upright position.

Clinical examination and endoscopy revealed a large exophytic mass at the base of the tongue extending to the right, in contact with the lingual surface of the epiglottis, which caused oropharyngeal airway narrowing.

The patient was admitted for further management. Laboratory tests showed mild leukocytosis (Table 1).

**Table 1:** Laboratory findings

Test	Patient value	Reference range
White blood cell count (WBC)	12.750	4.000-11.000
Neutrophils (Neu)	71.25	40-75
C-reactive protein (CRP)	14.1	0-5

A magnetic resonance imaging (MRI) identified a 4.9 × 4.3 × 4.2 cm space-occupying lesion at the tongue base, displacing the uvula and extending to the epiglottis, with jugular chain lymph nodes up to 1.1 cm (level IIa).

**Figure 1:**
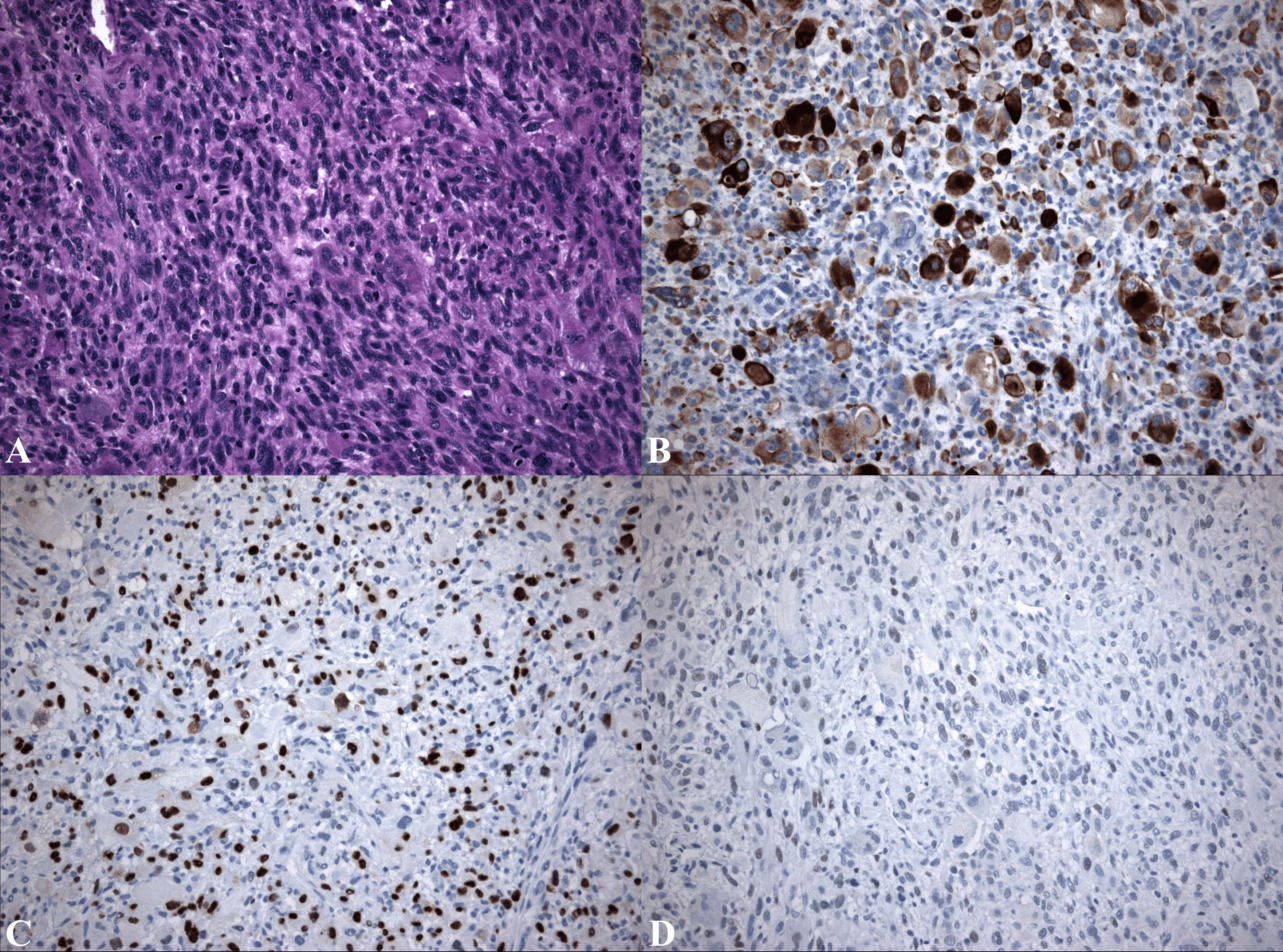
Histological findings A) Sheets of pleomorphic cells (including cells with rhabdoid features) with frequent mitoses, H&E 200×. B) Strong desmin immunopositivity, desmin 200×. C) Strong focal immunostaining for myogenin, myogenin 200×. D) Weak focal immunopositivity for MyoD1, MyoD1 200×.

Histology showed a pleomorphic neoplasm consisting of large, atypical, spindle, and rhabdoid cells. The tumor cells were diffusely immunopositive for desmin and focally for myogenin, whereas MyoD1 showed focal weak immunopositivity (Figure 1).

**Figure 2:**
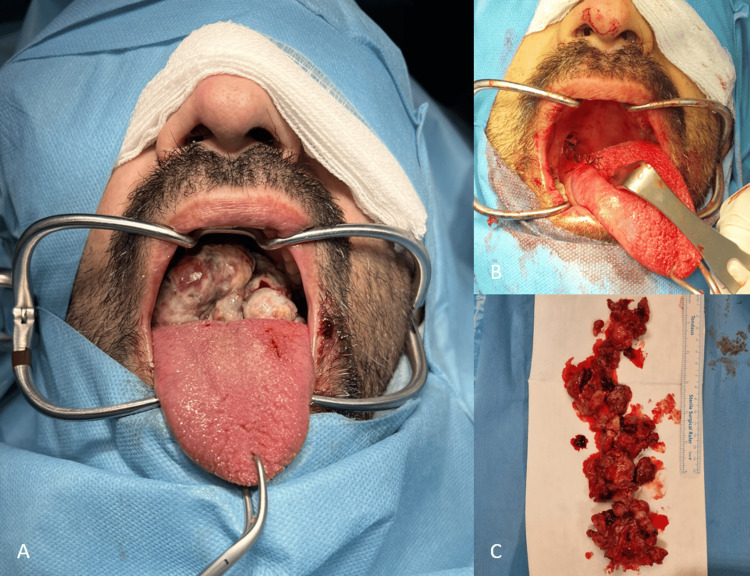
Intraoperative findings A) A large lesion originating from the base of the tongue is visible. B) Postoperative view of the mouth and tongue after lesion removal. C) The excised lesion.

Due to deteriorating symptoms and mild respiratory distress, the patient underwent surgical resection, with the official histopathology report of the initial biopsy pending. After nasotracheal intubation and tracheostomy, a mouth gag was placed, and the tongue was retracted anteriorly, exposing the tumor at the right tongue base. Critical attention was paid to identifying and preserving the lingual nerve, lingual vessels, and the hypoglossal nerve, which were in close proximity to the deep margin of the lesion. Dissection was performed with nerve monitoring. The mass, originating from intrinsic tongue muscles, was excised with 1.5 cm margins. The tongue was sutured with absorbable Vicryl sutures, and a nasogastric feeding tube was placed (Figure 2).

The postoperative course was uneventful. On postoperative day 5, fiberoptic endoscopic evaluation of swallowing confirmed safe swallowing without aspiration or penetration. The nasogastric and tracheostomy tubes were removed, and the patient was discharged on postoperative day eight with follow-up instructions.

**Figure 3:**
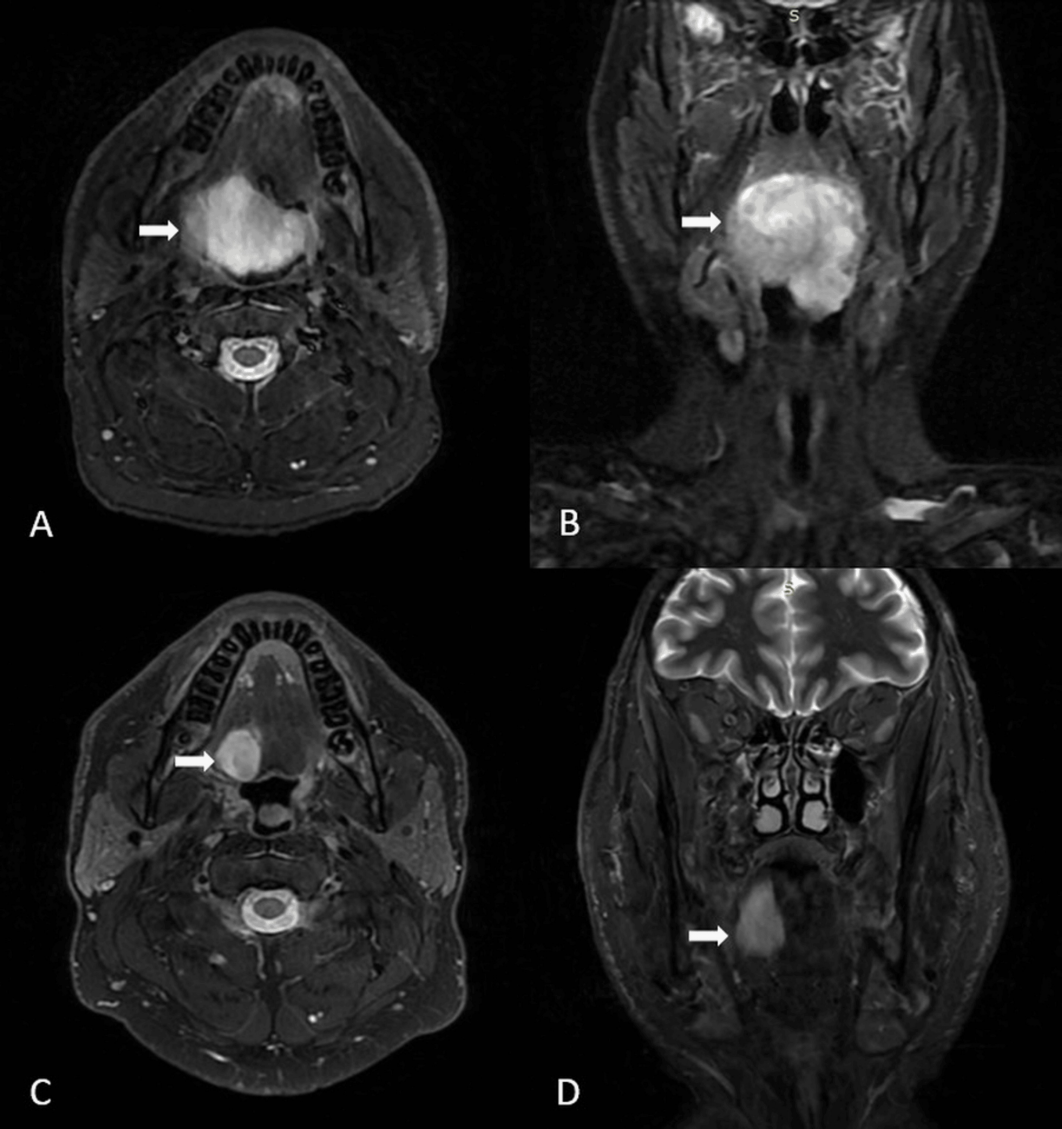
Imaging findings (white arrow showing the lesion) A,B) Axial and coronal T2-weighted MRI showing a preoperatively hyperintense lesion at the tongue base, extending laterally. C,D) Axial and coronal T2-weighted MRI revealing a recurrent lesion on the right lateral aspect of the tongue.

The histopathological examination of the entire specimen and the initially taken biopsy were made available approximately six weeks after the operation. A pre-irradiation follow-up MRI of the neck revealed an oval-shaped lesion measuring 1.6 × 2 cm on the right lateral aspect of the tongue base, with homogeneous enhancement and diffusion restriction, consistent with tumor recurrence (Figure 3).

Six weeks after a second surgical resection, the patient received definitive external irradiation. At the 12-month follow-up, there was no evidence of recurrence.

## Discussion

RMS of the tongue in adults is an exceptionally rare malignancy, even within the head and neck or the oral and paraoral regions [7]. In their 1973-2007 analysis, Turner et al. identified only 21 cases of tongue RMS among 558 head and neck RMS cases, with adults comprising just 31.1% of the total patient cohort [3]. Other studies reporting the occurrence of RMS of the tongue either focus on pediatric cases rather than adults or do not provide complete data regarding the patient profile and treatment approach. Due to its rarity, available literature on adult cases remains limited, with most data deriving from case reports and small retrospective studies. To our knowledge, only 10 studies have reported managing tongue RMS [8-17]. These included 12 adults (11 males and one female), with ages ranging from 29 to 66 years (Table 2).

**Table 2:** Summary of reported cases of adult tongue rhabdomyosarcoma, including patient demographics, tumor characteristics, treatment modalities, and clinical outcomes

Authors	No	Age	Sex	Key findings
Díez-Montiel et al. [8]	1	46	M	Right half tongue RMS, partial glossectomy, and VAC chemotherapy, achieving disease-free survival at 42 months.
Jariod-Ferrer et al. [9]	1	29	M	Surgical excision plus chemotherapy and radiotherapy, four years disease-free
Hartmann et al. [10]	1	41	M	Spindle cell RMS, six-year survival after multimodal treatment, aggressive surgical intervention, and subsequent recurrences
Ortiz Requena et al. [11]	1	36	M	Left mid tongue RMS, surgical excision, and no evidence of disease recurrence over a 17-month follow-up period.
Baldi et al. [12]	1	65	M	Embryonal RMS treated with surgery and postoperative radiotherapy, resulting in a disease-free status at 48 months follow-up.
Knipe et al. [13]	1	66	F	Sclerosing base RMS can be effectively managed with surgical resection and adjuvant radiotherapy.
Owosho et al. [14]	1	33	M	Investigated the diverse clinical presentations and molecular characteristics of spindle cell and sclerosing RMS. Significant heterogeneity, emphasizing the importance of molecular profiling.
Doval et al. [15]	1	32	M	Posterior third tongue RMS, treatment with surgical resection plus adjuvant radiotherapy.
Agaimy et al. [16]	3	59	M	Intramuscular unique subset of spindle cell tongue RMS characterized by recurrent VGLL3 gene fusions, predominantly affecting the tongue. Treatment with glossectomy plus VAC chemotherapy or surgery alone.
		42	M	
		54	M	
Strauss et al. [17]	1	53	M	Right side tongue RMS, treated with surgical resection plus chemotherapy and radiation. No recurrence at 12-month follow-up.
Present case	1	42	M	Base tongue pleomorphic RMS treated with surgical resection plus radiation. Recurrence treated with aggressive surgical intervention. Twelve months disease-free.

Our findings are in line with previous studies on the diagnosis of tongue RMS (i.e., a palpable mass, accompanied by dysphagia, muffled voice, or airway obstruction). Imaging studies, including MRI or CT scans, are crucial for evaluating the tumor’s size, location, and extent of invasion, while positron emission tomography (PET)-CT scans may be utilized to assess potential metastases. Definitive diagnosis is established through biopsy and histopathological analysis, with immunohistochemical markers, including desmin, myogenin, and MyoD1, confirming rhabdomyoblastic differentiation. However, definitive diagnosis may be challenging, especially in the presence of comorbidities (i.e., neurofibromatosis type 2 in the present case), thereby potentially delaying definitive treatment.

Adult tongue RMS presents with different histological variants, which influence treatment strategies and patient outcomes [4-5]. Spindle cell RMS, reported by Hartmann et al., Owosho et al., and Agaimy et al., demonstrated significant molecular heterogeneity and often required aggressive surgical management [10,14,16]. Our case followed a similar course with these findings, as the patient required two surgical interventions within four months before undergoing radiation therapy. Sclerosing RMS, documented by Baldi et al., was effectively treated with surgery and radiotherapy [12]. Embryonal RMS, as described by Knipe et al., also responded well to surgical resection and radiotherapy [13]. Cases of unspecified RMS, including those reported by Díez-Montiel et al., Jariod-Ferrer et al., Ortiz Requena et al., Doval et al., and Strauss et al., were primarily managed with surgical excision, often combined with vincristine, actinomycin D, and cyclophosphamide (VAC) chemotherapy and/or radiotherapy, reiterating the importance of multimodal therapy in optimizing patient outcomes [8-9,11,15,17].

Surgical resection was the primary treatment in all reported cases of adult tongue RMS, including the present one. Additionally, two patients received VAC chemotherapy, while three underwent radiotherapy, and two received a combination of both, particularly for spindle cell and embryonal RMS, to reduce recurrence risk and improve survival. This multimodal approach underscores the necessity of integrating local tumor control with systemic therapy to achieve better long-term outcomes. That being said, however, we need to consider that chemotherapy is rarely sufficient for disease control on its own merit and in very selected cases, thus occasionally necessitating a second-look histopathological assessment, which may delay definitive treatment.

Survival rates for RMS show conflicting findings in the literature. Studies generally indicate that prognosis is slightly more favorable in children, with embryonal RMS having better outcomes than the more aggressive alveolar RMS [18-19]. By contrast, adults tend to have a poorer prognosis due to delayed diagnosis, aggressive tumor behavior, and limited treatment responses. In Curry et al.'s study, the five-year relative survival for head-neck pediatric cases was 73.2%, whereas Zarrabi et al. reported five-year survival rates ranging from 26.6% to 61% in adults, emphasizing poor outcomes across all age groups [19-20]. Turner et al. reported that patients with parameningeal tumors, including tongue RMS, were more likely to present with regional or distant spread [5]. However, the aforementioned studies did not exclusively focus on tongue RMS. In this review, most cases of adult tongue RMS reported disease-free survival, with follow-up periods ranging from 12 months (as in the presented case) to six years despite tumor recurrences in some instances [10]. Larger patient studies are needed to evaluate the long-term survival outcomes of adult tongue RMS.

## Conclusions

RMS of the tongue is a rare but aggressive malignancy in adults that requires a multimodal treatment approach. The presented case, along with the 12 previously reported patients, highlights, on the one hand, the importance of considering RMS in the differential diagnosis of adult patients presenting with tongue masses, but points out, on the other, the potential difficulties pertaining to a definite diagnosis. Early diagnosis and comprehensive management, including surgery, radiotherapy, and chemotherapy, are crucial for improving outcomes. Given the high risk of recurrence, regular follow-up is essential, often necessitating multiple surgical interventions to achieve long-term disease control.
